# Evaluation of Various Ejector Profiles on CO_2_ Transcritical Refrigeration System Performance

**DOI:** 10.3390/e24091173

**Published:** 2022-08-23

**Authors:** Anas F. A. Elbarghthi, Václav Dvořák

**Affiliations:** Department of Applied Mechanics, Faculty of Mechanical Engineering, Technical University of Liberec, Stdentská 1402/2, 46117 Liberec, Czech Republic

**Keywords:** ejector, power consumption, R744, COP, exergy destruction

## Abstract

This study examines the potential impact of the different ejector profiles on the CO_2_ transcritical cooling system to highlight the contribution of the multi-ejector in the system performance improvement. The research compares the implementation of an ejector-boosted CO_2_ refrigeration system over the second-generation layout at a motive flow temperature of 35 °C and discharge pressure of 90 bar to account for the transcritical operation mode. The result revealed a significant energy saving by reducing the input power to the maximum of 8.77% when the ejector was activated. Furthermore, the multi-ejector block could recover up to 25.4% of the expansion work losses acquired by both ejector combinations VEJ1 + 2. In addition, the behavior of the multi-ejector geometries and operation conditions greatly influence the system exergy destruction. The analysis shows a remarkable lack of exergy destruction during the expansion process by deploying the ejector in parallel with the HPV.

## 1. Introduction

In recent times, there has been a major global call to replace deleterious refrigerants with more eco-friendly alternatives. Alexander Twining first produced carbon dioxide (R744). It was officially recognized as a British patent and as being fit for use as a refrigerant in 1850. Over time, it has become one of the most popular refrigerants among many [1]; the Coca-Cola Company, for instance, is planning to discontinue the use of HFCs by adopting CO_2_ technology as a leading solution [2]. When R744 became known as a refrigerant, it was greeted by stiff skepticism and stern criticism among relevant scientific communities [3]. The reason for this was the characteristic heat sink operational pressure and low system efficiency, which call for another mechanism integration that fosters technical advancement and addresses unprecedented technological challenges. Recently, these challenges and the quest for system advancement have been addressed through better process design, which has shed more light on the merits of using CO_2_ in cooling systems.

It has been established that CO_2_ has superior thermal properties over other refrigerants. For instance, natural refrigerants represent the class with the highest thermal conductivity and specific heat capacity. They also possess the high latent heat of vaporization needed for a more efficient heat transfer within the evaporator. Moreover, CO_2_ features a high volumetric refrigeration capacity, which is well known to significantly influence the heat transfer coefficient [4]. In addition, CO_2_ has a low viscosity, which mainly reduces the initial investment cost due to the cost-effective geometrical properties of the valves, pipelines, and other ancillary components.

In contrast, CO_2_ has a high saturation pressure of 4–12 times that of other refrigerants, which requires special technical considerations during the manufacturing process. Thus, due to the merits of the excellent properties of natural refrigerants, they are a favorable choice of working fluid [4]. The market has witnessed a surge in the applications using this refrigerant in cooling systems worldwide. This is encapsulated in the fact that ejectors improve the performance of transcritical refrigeration cycles when integrated with parallel compressors to attain the attendant pressure lift. The advantages of using the ejector have resulted in many ground-breaking types of research in terms of significant energy reduction, which is greatly reliant on the ejector geometries, refrigerant properties, and the core purpose of its applications.

A large number of publications have presented experiments on the R744 ejector, which illustrates the impact of the inclusion of the ejector on transcritical systems. Elbel and Hrnjak reported improvements of up to 8% in the total system COP by using a prototype ejector in their experimental work [5]. In the same regard, 15% higher COP was reported experimentally when the ejector was operated, in comparison with the conventional base system [6]. Furthermore, Nakagwa et al. conducted research on the ejector-boosted system and experimentally demonstrated up to 27% higher COP [7]. In addition, further published research proved the possibility of enhancing the total system COP from 20% to 30% by using the ejector to recover the expansion work [8].

Hafner et al. [9] introduced a multi-ejector block containing different ejector cartridge geometries. This concept supports the activation of any profile combination in parallel to suit any requested capacity, keeps the work recovery at the optimum level, and accurately maintains the gas cooler pressure values. The multi-ejector strategy significantly improves several aspects of the refrigeration system. For example, four different ejector cartridges were tested by Banasiak et al. to map the performance of each profile separately and detect the greatest work recovery provided [10]. For the sake of optimization and to study the irreversibilities of the ejector, several computational and numerical works were performed to predict the influence of the ejector efficiency on the refrigeration cycle [11,12,13,14,15,16,17,18].

The impact of ejector geometries has been studied in many experimental studies [19]. For example, an adjustable ejector motive nozzle throat was tested by XU et al. [20]. The authors stated a 20–30% distribution of the ejector efficiency, which led to maximizing the system COP. In addition, fixed and adjustable parallel ejector arrangements were evaluated by Smolka et al. [21] to deliver a flexible mass flow. The results showed that the controllable-geometry design does not exceed 35% of the ejector efficiency, while the fixed geometry configurations can produce higher efficiency concerning the operating conditions.

Elbarghthi et al. implemented an extensive experimental study that used a small ejector throat to analyze the ejector performance under the subcritical and supercritical regions of operations [22]. The result revealed a high ejector efficiency that could allow 36.9% of the available work rate to be recovered and reach 23% of exergy efficiency at a high exit gas cooler temperature. Gullo et al. proved that a CO_2_ multi-ejector outperformed other fluorinated working fluids in conventional-based solutions, especially in northern and central Europe [23]. The results showed 26.9% higher energy savings in average-sized supermarkets utilizing CO_2_ as a refrigerant. The ejector has contributed to the air conditioning applications, and can reduce the total system power consumption by 8.3–8.6% in different system configurations.

Multi-ejector blocks use different cartridge combinations to recover the maximum available work in the system. One of the challenges in this field is to study the best receiver working conditions when different ejector cartridges are running because each cartridge has a limited pressure lift; otherwise, the malfunction mode will exist in operations at high receiver pressure, thus influencing the overall system performance. In this regard, this paper aims to examine the impact of using various ejector profiles in improving general system performance. The study compares the implementation of an ejector-boosted system over the second generation of CO_2_ transcritical refrigeration system. The performance of the ejectors and the overall system operational characteristics is emphasized as the main objective of the study.

The analysis covers different characteristics, such as the system COP, the exergy destruction, and the contribution of the ejector to the total input power reduction.

## 2. System Configuration

**Figure 1:**
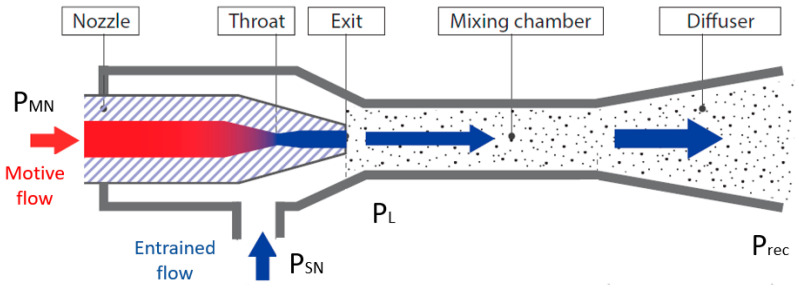
The schematic diagram of the ejector adapted from Danfoss.

**Figure 2:**
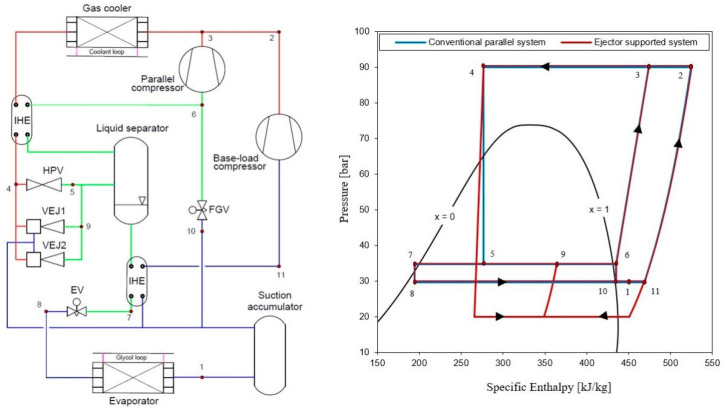
The schematic and P-h diagram for ejector-boosted R744 transcritical refrigeration system.

Figure 1 represents a simple ejector schematic diagram. The graphical R744 transcritical refrigeration system supported with an ejector used in the analysis is shown in Figure 2. The cooling cycle consists of a base-load compressor used to compress the expanded vapor from the low temperature and pressure region (evaporator pressure range) to the gas cooler pressure region. The system adopts a supplementary compressor indicated as a parallel compressor to extract the vapor from the liquid separator pressure level to the gas cooler pressure. In the calculations, Dorin semi-hermetic compressors, type CD1400H and CD380H, were used based on their polynomial functions that defined the mass flow rate and the power consumption provided by the supplier. However, this layout boosts the unloading of the base-load compressor. As a result, extra power is consumed by the parallel compressors, but the total input power from all the compressors in the system is reduced and the system COP is improved [24,25]. The refrigerant rejects the heat at the gas cooler using the glycol cycle, which serves as the heat sink and then leaves (state 4) to the liquid separator after expanding through HPV (state 5). From the receiver, the vapor portion is supplied to the parallel compressor suction line (state 6–3) while the liquid (state 7) is fed to the evaporator through the expansion valve device (state 8). This circulation mechanism has been proved to enhance the distribution of R744 in the cycle [26].

The exit gas cooler temperature is sensitive to the environment, and increases in a hot climate, providing a massive amount of the flashed gas, which can reach 50% of the entire mass flow in the system [27]. Nonetheless, this configuration represents the booster parallel refrigeration system, which is compared with the ejector-supported system where the ejector is connected in parallel with HPV for the overall system performance improvement evaluation.

In this study, two different ejector profiles, VEJ1 and VEJ2, are utilized. These ejector cartridges have been studied comprehensively in the literature, and their performances have been represented with reasonable accuracy in approximation functions [10,28]. The main geometries of both ejector profiles are listed in Table 1. When the ejector cartridges are on, a portion of the gas cooler exit working fluid passes through the primary ejector nozzle, which expands and generates a local pressure drop that allows the entrainment of the secondary flow stream from the evaporator exit (state 1). The mixed stream then passes to the liquid separator at a pressure higher than the suction flow pressure level (state 9).

The study was undertaken for a −6 °C evaporation temperature, and gas cooler pressure and temperature of 90 bar and 35 °C, respectively, to account for the transcritical operation. The proposed system cooling capacity was analyzed for 10 kW because the used cartridges are quite small and the goal was to indicate the significant benefit of these two ejector profiles on the system performance improvement.

## 3. System Performance Calculations

Many parameters are used to evaluate the two-phase expansion ejector performance, represented as the pressure lift, entrainment ratio, and ejector efficiency, and described as the ratio of the expansion work recovery to the maximum potential work. The entrainment ratio is calculated as the suction nozzle mass flow rate ratio to the motive nozzle, as represented in Equation (1). The best ejector performance can be achieved when a large pressure lift is obtained with a high suction mass flow rate. The pressure lift is defined as the pressure difference between the ejector outlet mixed stream and the suction nozzle pressure, as described in Equation (2). The ejector efficiency is calculated based on the derivation provided by Elbel et al. [5]. This formula expresses the ejector’s total irreversibility and has been used in many studies in the literature as a simple model for measuring efficiency because it relies on the operating boundary conditions of the ejector and no information is needed from the entire flow [29,30,31].

(1) $$\mathrm{ER}={ṁ}_{\mathrm{SN}}/{ṁ}_{\mathrm{MN}}$$

(2) $$P_{\mathrm{lift}}=P_{\mathrm{rec}}-{\;P}_{\mathrm{SN}}$$

(3) $$\eta_{\mathrm{ej}}={Ẇ}_{r}\;/\;{Ẇ}_{r,\max}$$

In Equations (1)–(3), ER represents the entrainment ratio; ${ṁ}_{\mathrm{SN}}$ and ${ṁ}_{\mathrm{MN}}$ are the suction mass flow rate and the motive nozzle mass flow rate, respectively, in kg/s; and $P_{\mathrm{rec}}$ is the liquid separator receiver pressure in bar, which characterizes the ejector outlet backpressure. $P_{\mathrm{SN}}$ represents the suction nozzle inlet pressure in bar. $\eta_{\mathrm{ej}}$ is the ejector efficiency. ${Ẇ}_{r}$ and ${Ẇ}_{r,\max}$ represent the actual recovered work of the ejector and the overall available work recovery potential, respectively, in kW. The calculations were developed using the first and second laws of thermodynamics based on the following constraints:

- the processes for all the analyses are steady-state;
- the pressure drop at the gas cooler, evaporator, and piping is not considered;
- the kinetic and the potential energies are neglected;
- the system is well isolated.

The main parameter used to evaluate the system is the coefficient of performance (COP), which describes the vantage cooling action provided to the energy input required, and is calculated as in Equation (4). The influence of the ejector, when integrated with the system, is evaluated by the determination of the COP improvement.

(4) $$\mathrm{COP}={\overset{\cdot }{Q}}_{\mathrm{evap}}\;/\;{Ẇ}_{\mathrm{comp}}$$

(5) $${\overset{\cdot }{Q}}_{\mathrm{evap}}={ṁ}_{{\mathrm{CO}}_{2}}\cdot \left({\;h}_{\mathrm{evap}.\mathrm{out}}-{\;h}_{\mathrm{evap},\mathrm{in}}\right)$$

(6) $${Ẇ}_{\mathrm{comp}}={Ẇ}_{\mathrm{comp}1}+{Ẇ}_{\mathrm{comp}2}$$

(7) $${\mathrm{COP}}_{\mathrm{improv}}=\frac{{\mathrm{COP}}_{\mathrm{ej}}-\;\mathrm{COP}}{\mathrm{COP}}\;\cdot 100\%$$

where ${Ẇ}_{\mathrm{comp}}$ is the compressors’ input power in kW and $Q_{\mathrm{evap}}$ is the cooling capacity in kW. Based on the second law of thermodynamics, the exergy destruction for the high-pressure valve in the cycle ${\overset{\cdot }{D}}_{\mathrm{HPV}}$ can be calculated by the specific exergy difference in any state, as revealed in Equations (8) and (9). For the exergy calculation, the environmental dead-state properties $T_{o}$ and $P_{o}$ were selected to be 20 °C and one atmospheric pressure.

(8) $$e_{i}=\left[h_{i}-h_{o}\right]-T_{o}\left(s_{i}-s_{o}\right)$$

(9) $${\overset{\cdot }{D}}_{\mathrm{HPV}}={ṁ}_{\mathrm{HPV}}\;\left(e_{\mathrm{in}}-e_{\mathrm{out}}\right)$$

## 4. Results and Discussion

### 4.1. Ejector Characteristic Functions

**Figure 3:**
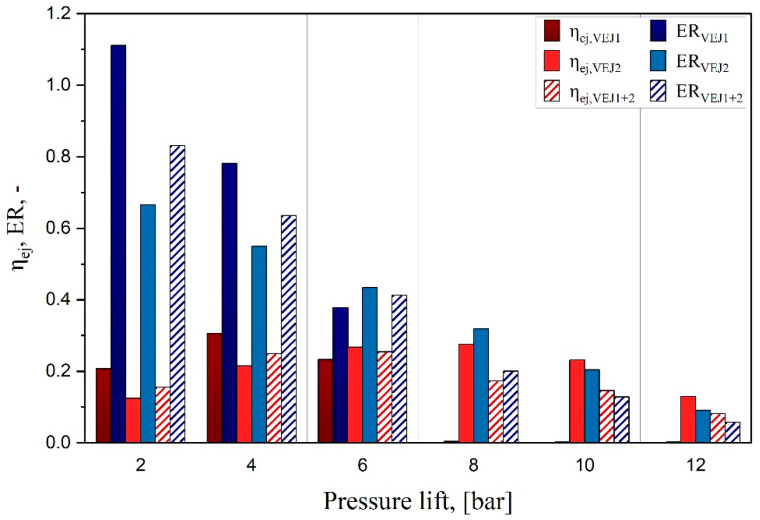
The ejector profile characteristics vs. pressure lift.

The results provide an in-depth comparison of utilizing the two ejector profiles with the parallel compressor system known in the literature as the second-generation layout. To test the influence of the ejectors on the system, the performance of these two ejectors should be illustrated and carefully tested to evaluate their efficiency and main driving characteristics. Figure 3 represents the characteristics of both ejector profiles and the combinations (VEJ1 + VEJ2), which depend on the pressure lift ranging from 2 to 12 bar with a step of 2 bar. The research was carried out based on an inlet motive nozzle temperature of 35 °C and exit gas cooler pressure of 90 bar considering the transcritical operation mode. The results revealed similar shortcomings exhibited by the ejector mass entrainment ratio when the pressure lift increases. For instance, increasing the liquid separator receiver pressure for a high-pressure lift creates shock waves inside the ejector, moving it closer to the motive exit position where it disturbs the flow with the less-entrained suction flow. Subsequently, the entrainment ratio drops. It should be noted that the small ejector cartridge provides a higher entrainment ratio compared to VEJ2 when the system is operating at a low-pressure lift; this is associated with a higher motive mass flow rate. Furthermore, the efficiency of the ejector of the VEJ1 recorded an optimum value of 31% under test conditions, whereas this profile experimentally registered higher efficiency of up to 37% [22] for different operational parameters.

The multi-ejector concept introduced by Hafner et al. [9], which has the flexibility of using various cartridges connected in parallel to reach maximum capacity while maintaining a more efficient work recovery, has proven to be more viable than the single fixed geometry ejector, which is the smallest vapor ejector cartridge presented by Banasiak et al. [10]. Its 1 mm throat diameter (VEJ2) was combined with the current cartridge (VEJ1) to evaluate the system performance. The VEJ2 performance was tested with 400 investigation points to produce a qualitative resolution; then, the approximation function was introduced for the inlet mass flow rate with reasonable accuracy. As shown in Figure 3, the behavior of both ejector combinations (VEJ1 + 2) relies significantly on the pressure lift and the inlet motive nozzle temperature. The results revealed the same trends for the ejector mass entrainment ratio of the VEJ. For instance, when both cartridges are activated at P_lift_ = 2 bar, ER reaches 0.83, which is lower than when using VEJ1 alone. However, the multi-ejector allows entraining a 50% higher suction mass flow rate with the ejector combinations, but the motive mass flow rate also experiences a surge. When the pressure lift is increased, the ER drops gradually, which vanishes for VEJ1 from P_lift_ = 8 bar where this profile is introduced as a normal expansion valve. In contrast, VEJ2 continues to produce a higher pressure lift to 12 bar with ER of 0.091. However, the ejector efficiency for VEJ1 + 2 acquired an optimum value of 25.4% reporting lower efficiency than using the VEJ1 profile alone but extended to cover a wide range of the operational condition. In other words, the combination of the ejector cartridges greatly influenced the system’s performance by improving the work rate recovered. The results demonstrated an increase in the recovered work rate to a maximum of 0.198 kW and recorded a rate that was 2.2-times higher overall than that of the single VEJ1 used under the same operating conditions based on Equation (3). Generally, when the systems are running in the transcritical state, the amount of flash gas increases, which increases the maximum work recovery potential, thereby reducing the ejector efficiency.

**Figure 4:**
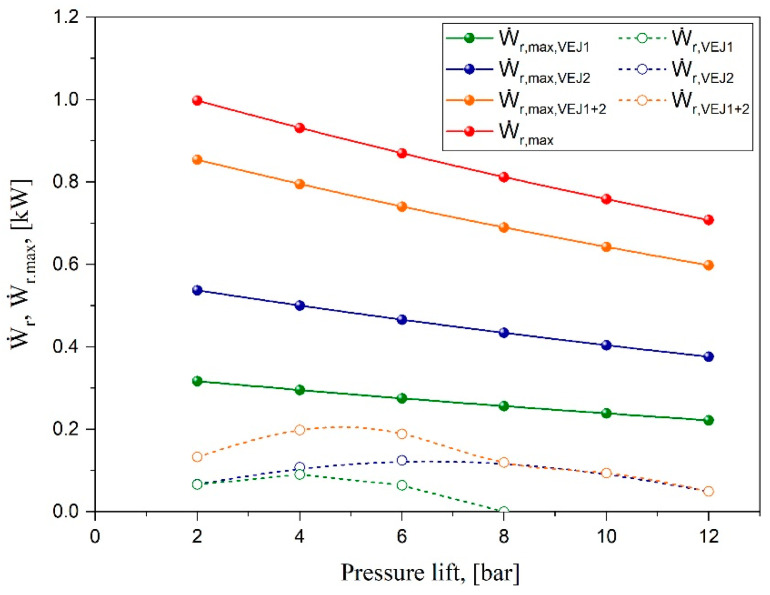
The work rate and maximum potential work recovery characteristics based on different ejector configurations and pressure lifts.

The most significant parameters that can be used to evaluate how the ejector can benefit the CO_2_ transcritical refrigeration systems are the expansion work rate recovery (Ẇ_r_) and the overall available work recovery potential (Ẇ_r,max_). These parameters indicate the power available to perform isentropic compression on the suction flow through the ejector to the separator and the maximum theoretical work recovery potential that depicts the total irreversibility of the ejector [22]. Figure 4 illustrates the work rate and maximum potential work recovery characteristics via different pressure lifts. The analysis was performed for the parallel compressor system layout as the baseline compared with varying configurations of ejectors for 10 kW cooling capacity. The results show the maximum work recovery rate of expansion in the high-pressure valve for the parallel system with expansion work ranging from 1 kW at P_lift_ = 2 bar to 0.7 kW when the pressure lift increases to 12 bar. This indicates the significant throttling loss of CO_2_ as a refrigerant compared with other low-pressure working fluids, especially at ambient temperatures that force the cycle to operate in transcritical mode. It can be seen that the smaller ejector cartridge VEJ1 could only recover up to 0.09 kW of the expansion work from the overall available work recovery potential of 0.3 kW. This ejector profile can only be used for a short range of liquid separator pressure with a pressure lift lower than 8 bar.

In contrast, the second ejector cartridge allows for recovery up to 0.13 kW, representing 27% of the overall available work recovery potential that this cartridge could provide. The reason for this is closely connected with the increase in the motive mass flow rate when the motive nozzle throat diameter becomes larger. Under similar operating conditions, VEJ2 proved to have 53% of the parallel system available work recovery potential. When both ejector cartridges are activated, the maximum available work recovery expands from 0.6 to 0.85 kW depending on the pressure lift, representing up to 86% Ẇ_r,max_ of the baseline system. Moreover, the multi-ejector block allows recovery of up to 0.2 kW of the expansion work, which represents 25.4% of the throttling losses according to the efficiency metrics and statistics. Therefore, this analysis is essential to map out each ejector’s performance and indicate the best range of operation conditions.

### 4.2. Ejector System Performance Improvement

**Figure 5:**
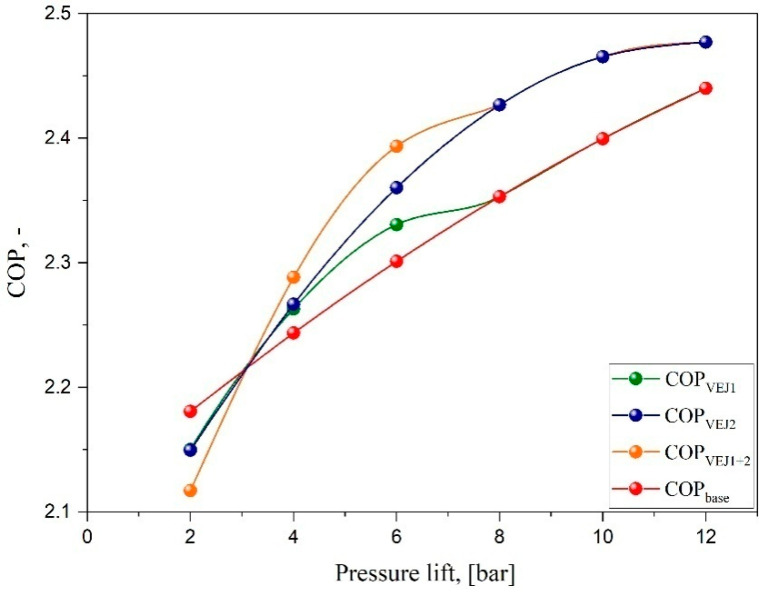
System COP characteristics vs. pressure lift for the booster system layout at different ejector configurations.

The impact of VEJ1 on system COP was experimentally tested for a wide range of operating conditions. However, implementing different ejector profiles for the transcritical CO_2_ cycle was determined for the system operational dynamics, including the COP, as shown in Figure 5. The results were obtained for the parallel compressor system and compared with different ejector configurations and pressure lifts. The outcome reveals that the COP has a proportional relation with the pressure lift. Increasing the separator pressure for a higher pressure lift provides a higher system COP based on the compression ratio reduction, decreasing the required input power and improving the performance. When the VEJ1 is activated, the system COP witnesses an increase of up to 1.2% compared with the baseline layout. It should be noted that the operation range for this cartridge is relatively short, which cannot benefit the system when the pressure lift exceeds 8 bar. By comparison, COP degradation was recorded when both ejector profiles ran at a pressure lift of less than 3.1 bar, despite operating with both ejector cartridges.

The influence of both ejector cartridges VEJ1 + 2 on the system performance is presented in Figure 5. The results indicate an appreciable improvement in the system COP obtained by running the multi-ejector to reach an optimum value of 2.39 at P_lift_ = 6 bar, representing a COP that is 4% higher than that of the booster system under the same working conditions. It can be noted that VEJ2 could support the system with a higher COP even for a pressure lift higher than 12 bar. It is also noteworthy that the system showed worse performance when operated at a low pressure lift compared to the booster baseline. The highest COP degradation was obtained at T_MN_ = 35 °C, P_lift_ = 2 bar for a value up to −2.9%. For this ejector configuration, the region of the COP improvement in the transcritical mode started at P_lift_ higher than 3.15 bar. In general, the multi-ejector block supported with more than one ejector profile can enhance the performance of the cooling system and meet any capacity needed by switching the required ejector electric solenoid valve.

**Figure 6:**
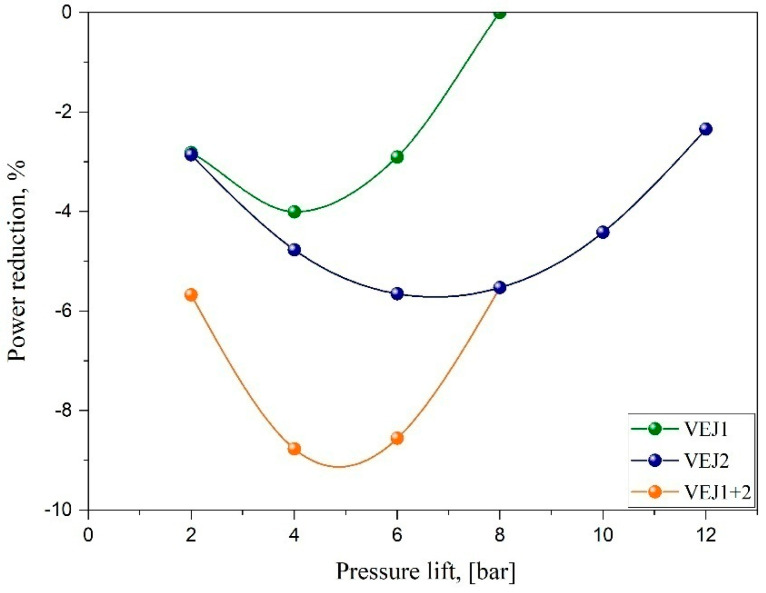
The impact of the ejector system on the compressor power recovery as a function of different pressure lifts.

The influence of the combination of the ejectors VEJ1 + 2 on the compressor power recovery for various pressure lifts is captured in Figure 6. The result depicts significant energy recovery achieved by reducing the compressor power by introducing the ejector profiles. For example, VEJ1 contributed to the reduction of up to 4% of the input power with respect to the exit gas cooler temperature at 35 °C. By comparison, implementing the ejector combination VEJ1 + 2 leads to the most significant power reduction. The maximum compressor power saving was 8.77% compared to operating the system in the absence of an ejector. It is also noteworthy that the total compressor power saving improved substantially to the minimum amount of 2.34% compared with the parallel layout depending on the ejector efficiency trend, which provided the optimum performance. In total, when VEJ2 is running, the input power is reduced by two to three times compared to running with a single ejector in VEJ1. This strategy indicates the advantages of operating with multi-ejector profiles where any requested capacity can be reached. In addition, a multi-ejector block is also able to control the discharge pressure and simultaneously maintain an efficient work recovery to a greater extent than other types of ejectors, such as the needle-based ejector.

**Figure 7:**
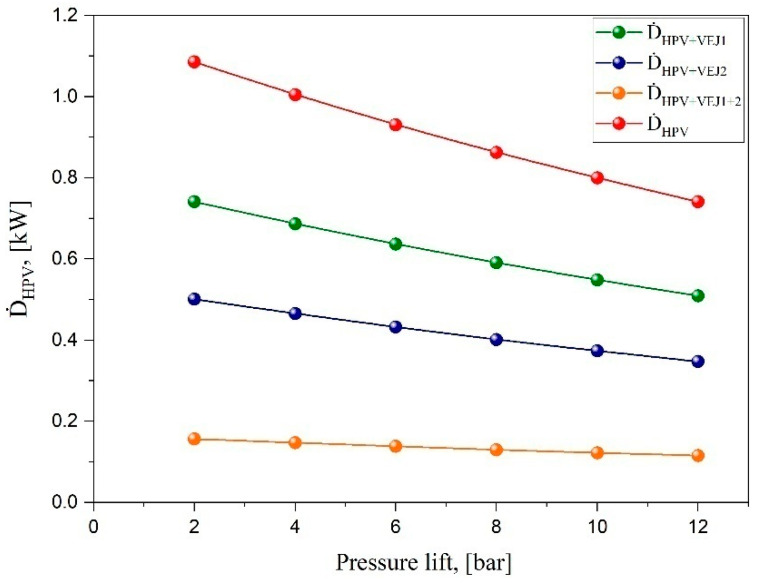
The HPV exergy destruction rate of the baseline parallel system and in the case of implementing different ejector profiles via different pressure lifts.

The exergy analysis is known by the maximum useful work, which can be determined at any thermodynamic state at equilibrium with the surroundings. CO_2_ transcritical refrigeration cycles exhibit remarkable throttling loss, which is recovered during the expansion process due to the significant difference in pressure between the heat rejected and the evaporation temperature. The expansion process takes place at the high-pressure valve. The ejector proved to be a reliable solution that could be connected in parallel with the HPV to recover the amount of work in question and improve the system performance. Figure 7 illustrates the HPV exergy destruction rate for the baseline parallel system compared with different ejector cartridge combinations at the variant level of pressure lift. The results revealed massive exergy destruction for the baseline system exceeding 1 kW at the operation level with low pressure lift. Increasing the pressure lift in operation provides a lower amount of irreversibility in the expansion process due to the reduction in the parallel pressure ratio of the compressors, which decreases the input power needed. However, when the small ejector profile of VEJ1 runs, the expansion process losses decrease by 31% compared to all operation ranges without an ejector, bringing the maximum exergy destruction to 0.74 kW. When the second ejector cartridge of VEJ2 runs alone with the HPV, the exergy destruction recues by 53%. The result indicates a significant improvement in the exergy destruction by using both cartridges together with the HPV. In total, more than 84% of the exergy losses during the expansion can be reduced by both ejectors. These results provide crucial energy savings for the CO_2_ refrigeration system operating at high ambient temperature and facing a high amount of flash gas in the second-generation layout of the transcritical systems.

## 5. Conclusions

The current study evaluated the impact of utilizing different ejector profiles on the performance of the R744 transcritical refrigeration system. The research ideas are premised on the first and second laws of thermodynamics. The approximation functions that experimentally described the performance of each ejector profile in previous work were implemented. The results were compared with the classical parallel layout as the baseline to reveal the contribution of ejectors to the recovery of the high irreversibilities during the expansion process, which reduces the exergy destruction. The most outstanding findings are summarized as follows:

- A total of 31% of available work was recovered by activating VEJ1, while the total efficiency acquired by both ejector combinations of VEJ1 + 2 registered an optimum value of 25.4%. However, the multi-ejector allows entraining a 50% higher suction mass flow rate with the ejector combinations, which greatly influences the system performance by improving the work rate recovered.
- CO_2_ transcritical refrigeration cycles possess significant throttling loss, especially at lower pressure lift values. In contrast, the combination of both ejector cartridges represented 85% of the potential work that the ejector implementation can achieve compared with the conventional layout.
- The multi-ejector concept was found to improve the overall system COP, which increased the refrigerating effect because a higher amount of liquid-phase refrigerant could be supplied to the evaporators. Moreover, the multi-ejector allowed pre-compression of the evaporator exit refrigerant prior to the intermediate pressure region and reduced the compressor input power needed to achieve this.
- In ejector technology, especially for those ejectors operating as supersonic ejectors in transcritical mode, the speed of sound and shock waves play a fundamental role and stand out as two crucial physical phenomena. They are responsible for choking flow and the increase in pressure inside the ejector. To consider the effects and dynamics of these parameters, an optimization CFD study should be performed to analyze these critical parameters.

## Figures and Tables

**Table 1:** The main geometry parameters of the ejectors.

Parameter name	Unit	VEJ1, [22]	VEJ2, [10]
Motive nozzle inlet diameter	mm	3.8	3.8
Motive nozzle diverging angle	degree	2	2
Motive nozzle converging angle	degree	30	30
Diffuser diameter	mm	7.3	7.3
Diffuser angle	degree	5	5
Motive nozzle throat diameter	mm	0.71	1.00
Motive nozzle outlet diameter	mm	0.78	1.12

## Data Availability

All data are available from the authors on request.
